# Flecainide toxicity with pill-in-pocket approach from accidental overdose: a case report

**DOI:** 10.1093/ehjcr/ytae522

**Published:** 2024-09-24

**Authors:** Mohamad Anas Oudih, Avraham Ginsburg, Mumin Hakim, Fengwei Zou, Nils Guttenplan

**Affiliations:** Department of Internal Medicine, Montefiore Medical Center-Wakefield Campus, 600 E 233rd St, Bronx, NY 10466, USA; Department of Emergency Medicine, St. John’s Riverside Hospital, 967 N Broadway, Yonkers, NY 10701, USA; Department of Emergency, Jacobi Medical Center, 1400 Pelham Pkwy S, Bronx, NY 10461, USA; Division of Emergency, Montefiore Medical Center, 111 E 210th St, Bronx, NY 10467, USA; Division of Electrophysiology, Department of Cardiology, Montefiore Medical Center, 111 E 210th St, Bronx, NY 10467, USA; Division of Electrophysiology, Department of Cardiology, Montefiore Medical Center, 111 E 210th St, Bronx, NY 10467, USA

**Keywords:** Flecainide toxicity, Pill-in-pocket, Arrhythmia, Atrial fibrillation, Wide complex tachycardia, Case report

## Abstract

**Background:**

The Pill-in-the-Pocket (PiP) approach may be used in highly selected patients to achieve acute pharmacological cardioversion into sinus rhythm. Flecainide toxicity is rarely reported, especially with patients who take flecainide as PiP, and only limited evidence exists in its management. We present a case of accidental flecainide overdose for a patient who is on PiP and the acute management strategy.

**Case summary:**

A 78-year-old female with persistent atrial fibrillation (AF), previously underwent pulmonary vein isolation and maintained on verapamil 240 mg twice daily, presented to the electrophysiology clinic following a recent hospital admission for recurrent AF. Due to infrequent recurrent episodes of symptomatic AF, the patient preferred to avoid both repeat ablation and additional daily medications. After an initial trial on telemetric monitoring, a PiP approach with flecainide 300 mg was adopted. Unfortunately, palpitations and dyspnoea in the context of chronic obstructive pulmonary disease exacerbation led the patient to self-medicate with multiple doses of albuterol and flecainide. Twelve-lead electrocardiogram showed slow AF with a wide QRS complex. The patient received 1 g of calcium gluconate with a 180 mEq bolus of sodium bicarbonate 8.4% and was started on continuous sodium bicarbonate infusion at 150 mL/h. Over a 12 h period, the QRS complex narrowed down, and the rhythm returned to normal sinus rhythm with a QRS interval of 136 ms.

**Discussion:**

The PiP strategy with flecainide is safe and effective. Reinforcement of medication dosing and frequency with patient read back is key to avoid accidental toxicity, which could be life-threatening. Treatment with sodium bicarbonate is quick and highly effective.

**Learning points**

- Reinforcement of the maximum dose and frequency of flecainide with patients who are initiated on the Pill-in-the Pocket approach is critical to avoid accidental toxicity.
- Sodium bicarbonate infusion is a safe and effective management strategy for flecainide toxicity.
- Patients who are maintained on flecainide and present with drug toxicity must be screened for structural heart disease and ischaemic cardiomyopathy.

## Introduction

Flecainide is an effective Class IC rhythm control agent for atrial fibrillation (AF) in patients without structural and ischaemic heart disease.^1,2^ The Pill-in-the-Pocket (PiP) approach can be used in highly selected patients to achieve acute pharmacological cardioversion into sinus rhythm.^3^ Flecainide overdose can cause life-threatening arrhythmias, and only limited evidence on flecainide toxicity management is available. We present a case of accidental flecainide overdose and its acute management.

## Summary figure

**Table:** 

25 October 2022	Admitted to hospital with atrial fibrillation. Cardioversion with flecainide was done under telemetric monitoring. The patient was discharge on verapamil 120 mg b.i.d. and flecainide 150 mg b.i.d.
11 November 2022	Follow-up visit: The patient preferred to avoid repeat ablation and additional daily medication. Pill-in-the-Pocket (PiP) approach with flecainide 300 mg as needed and verapamil 120 mg twice daily was adopted.
6 November 2023 (5 a.m.)	Presented to emergency with generalized weakness. ECG showed atrial fibrillation with a heart rate of 80 and a wide QRS complex of 400 ms and QTc of 641 ms.
6 November 2023 (6 a.m.)	Toxicology recommended to start sodium bicarbonate infusion.
6 November 2023 (4 p.m.)	QRS interval narrowed to 136 ms on serial ECGs. Sodium bicarbonate infusion stopped as QRS duration became 136 ms and pH reached 7.6.
7 November 2023	Echocardiography showed newly reduced left ventricular ejection fraction and akinetic left ventricular basal inferior wall.
13 November 2023	After medical optimization: Left heart catheterization showed severe ostial stenosis of the right coronary artery and moderate to severe stenosis in the proximal to the middle segment of the left anterior descending artery. The patient had a drug-eluting stent placed in the proximal RCA.
26 November 2023	Admitted for asthma exacerbation. Was found to have rapid atrial fibrillation, which was treated with metoprolol and digoxin. After discussion with the patient, she agreed to repeat the ablation procedure.
24 January 2024	Repeat ablation: A reconnection of the right superior pulmonary vein, which was re-isolated at the right carina. Isolation of the coronary sinus and superior vena cava was performed.

## Case summary

**Figure 1:**
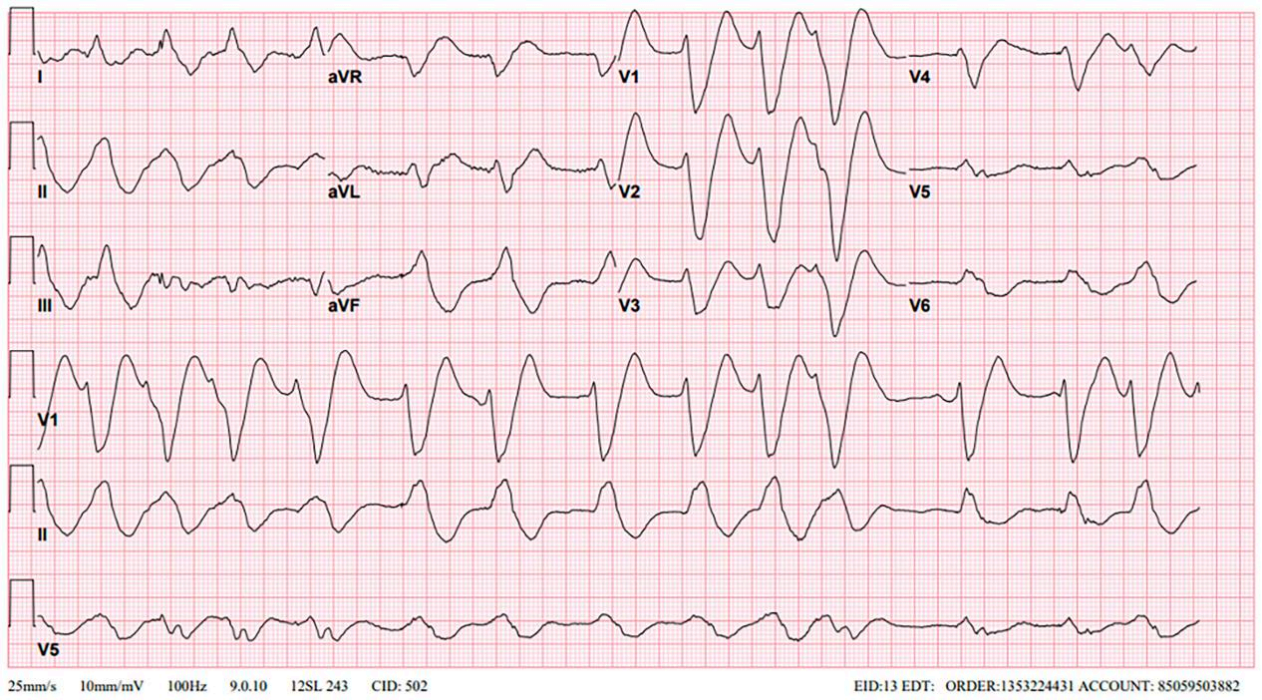
Atrial fibrillation rhythm with ventricular rate of 80 b.p.m., PR interval indetermined, QRS duration 400 ms, QTc interval 641 ms.

A 78-year-old woman with persistent AF, previously underwent pulmonary vein isolation and maintained on verapamil 240 mg twice daily, presented to the electrophysiology clinic following a recent hospital admission for recurrent AF. During that admission, an echocardiogram revealed no valvular or structural abnormalities, and a recent nuclear stress test showing normal myocardial perfusion. Given these findings, the patient underwent successful cardioversion with 300 mg flecainide orally under telemetric monitoring. The patient’s baseline electrocardiogram (ECG) showed normal sinus rhythm with PR interval of 140 ms, QRS duration of 76 ms, and QTc duration of 436 ms. Her ECG after initiating flecainide showed sinus rhythm with PR of 202 ms, QRS of duration 134 ms, and QTc of 436 ms. The patient was discharged on flecainide 150 mg twice daily. During follow-up, discussions for rhythm control options including continue antiarrhythmics or repeat ablation were introduced. However, the patient preferred to avoid repeat ablation at this time and a PiP approach with flecainide 300 mg as needed with daily verapamil 120 mg twice daily was adopted. The patient remained in sinus rhythm without complications on this regimen for one year with close follow-up. Unfortunately, asthma exacerbation and frequent albuterol use led the patient to self-medicate flecainide whenever her heart rate was higher than 100 on the home pulse oximeter. She ended up taking flecainide 900 mg daily for 7 days. Shortly after that, the patient presented to the emergency department with complaints of generalized weakness, nausea, vomiting, and shortness of breath. She was haemodynamically stable, and blood workups were troponin I < 0.01 ng/mL (normal < 0.3 ng/mL), creatine kinase 124 U/L (normal < 200 U/L), B-type natriuretic peptide 543 pg/mL (normal < 250 pg/mL), creatinine 1.38 mg/dL (<1.20 mg/dL), baseline 0.82 mg/dL, serum pH (7.39), bicarbonate (23 mEq/L), and completed blood count and electrolytes were normal. Twelve-lead ECG showed AF with a wide QRS complex and ventricular rate of 80 b.p.m. (*Figure 1*).

**Figure 2:**
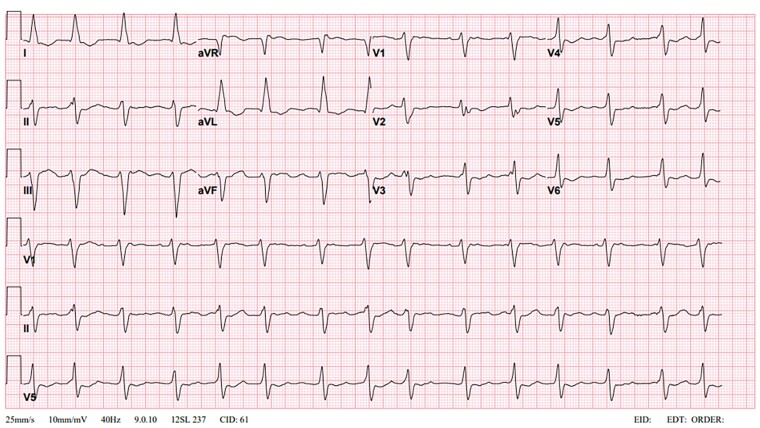
ECG after 8 h of admission and after 2 h of initiating sodium bicarbonate infusion showing atrial fibrillation rhythm with a ventricular rate of 90 b.p.m. QRS duration narrowed down to 184 ms.

**Figure 3:**
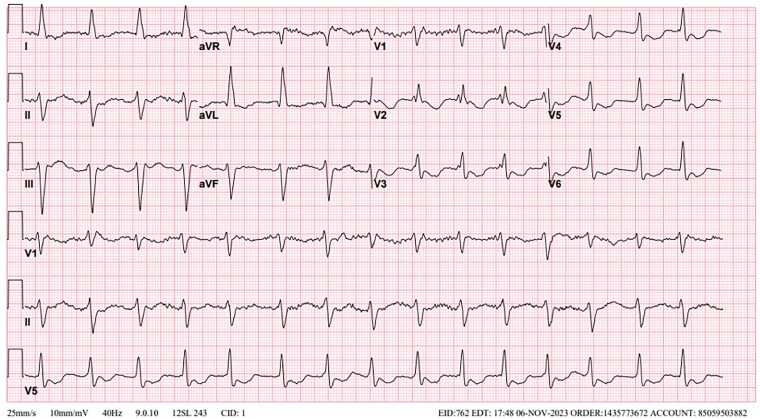
ECG after 10 h of admission and after 4 h of initiating sodium bicarbonate infusion. Sinus rhythm has replaced atrial fibrillation with a ventricular rate of 91 b.p.m. and QRS duration of 136 ms.

The patient was admitted to the cardiac care unit, and after consultation with toxicology, a trial of 180 mEq bolus of sodium bicarbonate 8.4% was given with one gram of calcium gluconate. A continuous sodium bicarbonate infusion at 150 mL/h was maintained for 12 h, and the QRS duration narrowed as serum pH rose (*Figures 2, 3*). Sodium bicarbonate infusion was stopped once pH crossed 7.60, and rhythm returned to normal sinus rhythm with a QRS duration of 136 ms, which is longer than baseline (80 ms).

While admitted, the patient underwent further cardiac workup. The echocardiogram revealed a newly reduced left ventricular ejection fraction and akinetic left ventricular basal inferior wall. Left heart catheterization showed a severe ostial stenosis of the right coronary artery (RCA) and moderate to severe stenosis in the proximal to middle segment of the left anterior descending artery (LAD). The patient had a drug-eluting stent placed in the proximal RCA with a plan for a staged percutaneous intervention of the LAD after discharge. After medical optimization, the patient was continued on a rate-control approach as well as guideline-directed medical therapy for heart failure, including losartan, empagliflozin, and spironolactone. She was discharged home with outpatient follow-up but returned with asthma exacerbation and was found to have rapid atrial fibrillation. After further discussion with the patient, she agreed to repeat the ablation and was scheduled for the procedure after discharge. On repeat ablation, the patient was found to have reconnection of the right superior pulmonary, which was re-isolated at the right carina. Moreover, isolation of the coronary sinus and superior vena cava was also performed. The patient was discharged 1 day following the procedure on an amiodarone 200 mg daily. During a follow-up appointment one month later, she reported experiencing another episode of AF within the blanking period. Consequently, the amiodarone dosage was increased to 200 mg twice daily for the duration of the blanking period.

## Discussion

Flecainide is a Class 1C antiarrhythmic agent that acts as a sodium channel blocker. Flecainide’s primary mechanism of action is on the action potential of Purkinje fibres and ventricular muscle by reducing the maximum upstroke slope of phase 0. The blocking effect of sodium channels depends mainly on the frequency of stimulation. The drug dissociation from the sodium channel is extremely low compared to other sodium channel blocker agents, which causes slow recovery and marked depression of the maximum upstroke slope of phase 0 (Vmax) with lengthening of the QRS duration. Flecainide shortens the duration of the action potential and effective refractory period in Purkinje fibres but prolongs them in atrial and ventricular muscle fibres by inhibiting IKr channels and delaying potassium rectifier current. Its main electrophysiologic action is marked by the prolongation of AH (the conduction time through the AV node), HV (the conduction time from the His bundle to the ventricular myocardium), PR, and QRS intervals.^1,2^

Flecainide is recommended as one of the first-line oral agents to achieve pharmacological cardioversion and maintain sinus rhythm in patients with AF and supraventricular tachycardias with a maximum dose of 400–450 mg/day.^3^ Its use is also indicated for the management of refractory ventricular arrhythmia.^4^ Furthermore, flecainide has proved effective in patients with catecholaminergic polymorphic ventricular tachycardia, where it helps reduce exercise-induced ventricular arrhythmias when used as an intensified treatment strategy.^5^

Flecainide is contraindicated in patients with structural heart disease and coronary artery disease (CAD). It has a proarrhythmic effect and can induce life-threatening arrhythmias in these patients. The CAST trial studied the effect of encainide and flecainide on the suppression of ventricular ectopy after myocardial infarction. It showed a significantly greater number of deaths and cardiac arrests due to arrhythmia, cardiac causes, or any cause than patients who received a placebo. The relative risk (RR) of death or cardiac arrest due to arrhythmia in patients receiving flecainide or encainide compared to placebo was 2.64, and the RR of death and cardiac arrest due to all causes was 2.38.^6^ Additionally, it is associated with a decrease in cardiac output and ejection fraction and an increase in pulmonary capillary wedge pressure.^1,3^

A recent retrospective analysis indicated that patients with obstructive CAD treated with Class IC agents had worse outcomes compared to those on Class III agents in terms of higher 30-day mortality rates, increased occurrences of ventricular arrhythmias, and more frequent admissions for decompensated heart failure. In contrast, Class IC and Class III agents exhibited a similar safety profile in patients with non-obstructive CAD. Additionally, there was no significant interaction between the use of Class IC agents and a diagnosis of systolic or unspecified heart failure.^7^

The PiP approach with flecainide is a feasible and safe approach for patients who suffer from recurrent AF and demonstrates a high compliance rate. It proved a low rate of adverse events and a marked reduction in emergency visits and hospital admissions.^8^ However, flecainide toxicity is life-threatening. To mitigate the risk of overdose, particularly in patients utilizing the PiP strategy, reinforcement of medication dosing and frequency with patient readback should be done every follow-up. Particular attention should be paid to patients with asthma or chronic obstructive pulmonary disease who are on chronic β1-agonist therapy, as it is crucial to differentiate between asthma exacerbation and genuine palpitation, which can lead to inappropriate use of flecainide.

Flecainide toxicity is a consequence of the sodium and calcium channel blockade effect on atrial and ventricular myocytes and the His-Purkinje fibres. The cardiac manifestations are caused by slowing the atrioventricular conduction and prolonging the refractory period in ventricular myocytes with a negative ionotropic action.^1,2,9^ The clinical features of flecainide toxicity include bradycardia, hypotension, and nodal and ventricular arrhythmias.^1,2,6^ The primary ECG abnormalities are prolongation of the QRS and QT intervals. Additionally, Brugada pattern has been reported in the literature. Other non-cardiac features occur secondary to impaired tissue perfusion, which includes hypoxia, metabolic acidosis, impaired kidney function, seizure, and the risk of respiratory depression.^10,11^ In our patient, significant QRS prolongation was observed, which is a life-threatening condition that requires emergent therapy.

Flecainide and other Class IC antiarrhythmic drugs remain contraindicated in patients with a history of myocardial infarction and ventricular arrhythmias. The possible interaction of flecainide with ischaemic and healed tissue enhances a patient’s vulnerability to drug-induced ventricular arrhythmias.^5^ Our patient was found to have previously unrecognized coronary artery disease with potential ischaemic cardiomyocytes, which could increase her susceptibility to flecainide toxicity. Flecainide toxicity is rarely reported, and only limited evidence exists in its management. Once QRS prolongation is detected, sodium bicarbonate should be initiated. Rapid boluses of 50–100 mL of 8.4% sodium bicarbonate have been shown to reduce QRS duration. During treatment with sodium bicarbonate, QRS duration, electrolytes, and pH must be monitored closely to maintain serum pH between 7.5 and 7.55.^12^ Our patient received boluses of sodium bicarbonate, but without a good response, so continuous bicarbonate infusion was initiated, which reduced the QRS duration to over 12 h. Another treatment associated with favourable outcomes is intravenous lipid emulsion (ILE) therapy. It has been suggested that the ILE can act as a ‘lipid sink,’ removing lipid-soluble drugs such as flecainide and limiting further interaction with sodium channels on cardiac myocytes.^13^ Additionally, for patients who do not respond to medical therapy or develop haemodynamic instability, mechanical circulatory support with extracorporeal membrane oxygenation or an intra-aortic balloon pump successfully stabilizes the patient until the drug is metabolized.^14^

## Conclusion

The use of flecainide as a Pill-in-the Pocket approach is effective for controlling atrial tachyarrhythmias. However, it must be initiated after thorough screening to rule out structural heart disease. Reinforcement of medication dosing and frequency with patient readback is critical to avoid accidental toxicity, which could be life-threatening. Treatment with sodium bicarbonate is quick and highly effective. Patients must be screened for structural heart disease and ischaemic cardiomyopathy before initiating flecainide. Moreover, it should be investigated as a possible cause of toxicity for patients who have been maintained on flecainide.

## Lead author biography


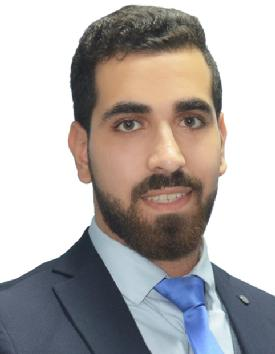


An internal medicine resident planning to pursue his career in electrophysiology with a strong commitment to integrating clinical practice with research. Dedicated to addressing health disparities, I aim to enhance cardiovascular care for underserved populations, blending rigorous research with compassionate patient care to make a meaningful impact in the field.

## Data Availability

The data used to support the findings of this case report are included within this manuscript.

## Notes

**Consent:** Patient’s written consent has been obtained before writing and submitting this manuscript, in accordance with COPE guidelines.

**Funding:** This research did not receive any specific grant from funding agencies in the public, commercial, or not-for-profit sectors.
